# The Metastability of the Double-Tripod Gait in Locust Locomotion

**DOI:** 10.1016/j.isci.2019.01.002

**Published:** 2019-01-08

**Authors:** Eran Reches, Daniel Knebel, Jan Rillich, Amir Ayali, Baruch Barzel

**Affiliations:** 1 Department of Mathematics, Bar-Ilan University, Ramat-Gan 52900, Israel; 2 School of Zoology, Faculty of Life Sciences, Tel-Aviv University, Tel-Aviv 69978, Israel; 3 Sagol School of Neuroscience, Tel-Aviv University, Tel-Aviv 69978, Israel

**Keywords:** Entomology, Evolutionary Ecology, Biomechanics

## Abstract

Insect locomotion represents a fundamental example of neuronal oscillating circuits generating different motor patterns or gaits by controlling their phase coordination. Walking gaits are assumed to represent stable states of the system, often modeled as coupled oscillators. This view is challenged, however, by recent experimental observations, in which *in vitro* locust preparations consistently converged to synchronous rhythms (all legs oscillating as one), a locomotive pattern never seen *in vivo*. To reconcile this inconsistency, we developed a modeling framework to capture the trade-off between the two competing mechanisms: the endogenous neuronal circuitry, expressed *in vitro*, and the feedback mechanisms from sensory and descending inputs, active only *in vivo*. We show that the ubiquitously observed double-tripod walking gait emerges precisely from this balance. The outcome is a short-lived meta-stable double-tripod gait, which transitions and alternates with stable idling, thus recovering the observed intermittent bouts of locomotion, typical of many insects' locomotion behavior.

## Highlights

• Isolated *in vitro* locust preparations indicate that idling is a stable fictive gait
• This is in contrast to the dominant *in vivo* locomotive pattern (i.e., double tripod)
• Hence functional locomotion behavior is dependent on descending and sensory inputs
• The presented model generates intermittent double-tripod bouts as seen empirically

## Graphical Abstract

**Figure:**
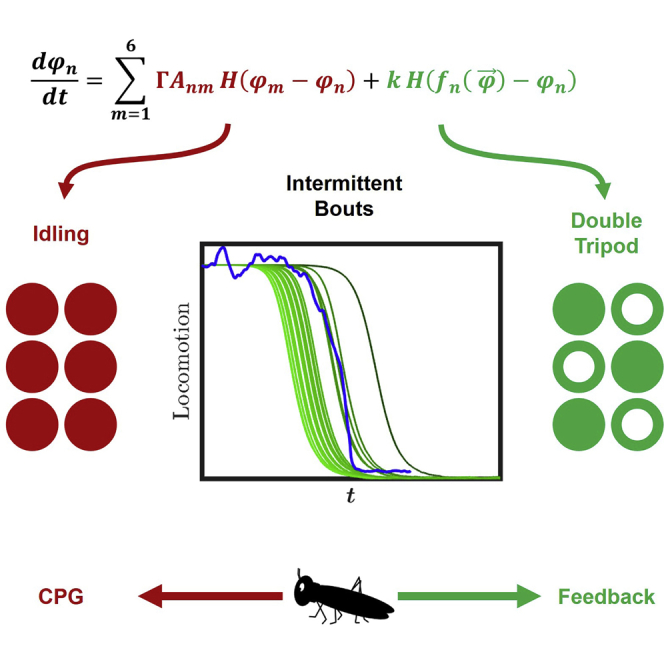

## Introduction

Six-legged locomotion is exceptionally effective, making, together with other traits, the insect family one of the most successful groups of organisms. One reason for this prominence is their remarkable capacity for dynamic stability: insects can rapidly generate adaptable movement in changing environments, employing multi-level adaptations while incorporating adaptive control mechanisms (Aminzare et al., 2018, Ayali et al., 2015b, Graham, 1985, Ritzmann and Büschges, 2007). Such locomotion patterns are driven by the insects' central nervous system, specifically its thoracic ganglia, which contains the basic circuitry for generating movement via networks of central pattern generators (CPGs) (Arshavsky, 2003, Bucher, 2009, David et al., 2016, Hooper and Weaver, 2000, Marder and Bucher, 2001, Marder and Calabrese, 1996). The movement is further coordinated through dynamic interactions between the central nervous system and sensory inputs from the rest of the body and the environment (Ayali et al., 2015a, Borgmann et al., 2009, Büschges et al., 2011, Friesen and Cang, 2001, Fuchs et al., 2011, Puhl and Mesce, 2010, Skinner and Mulloney, 1998, Yu and Friesen, 2004, Zill et al., 2009), as well as descending inputs from the head ganglia that mediate initiation, maintenance, and modification of locomotion motor patterns (Bender et al., 2010, Gal and Libersat, 2006, Guo and Ritzmann, 2013, Kien, 1990a, Kien, 1990b, Kien and Altman, 1984, Kien and Williams, 1983, Knebel et al., 2019, Martin et al., 2015, Mu and Ritzmann, 2008, Ridgel and Ritzmann, 2005). The relative importance of these different complementary components, central versus descending and sensory, in generating adaptable locomotion behavior is still largely an open question (Ayali et al., 2015a, Ayali et al., 2015b, Büschges et al., 2011, Cruse, 2002, Knebel et al., 2017, Mantziaris et al., 2017).

**Figure 1:**
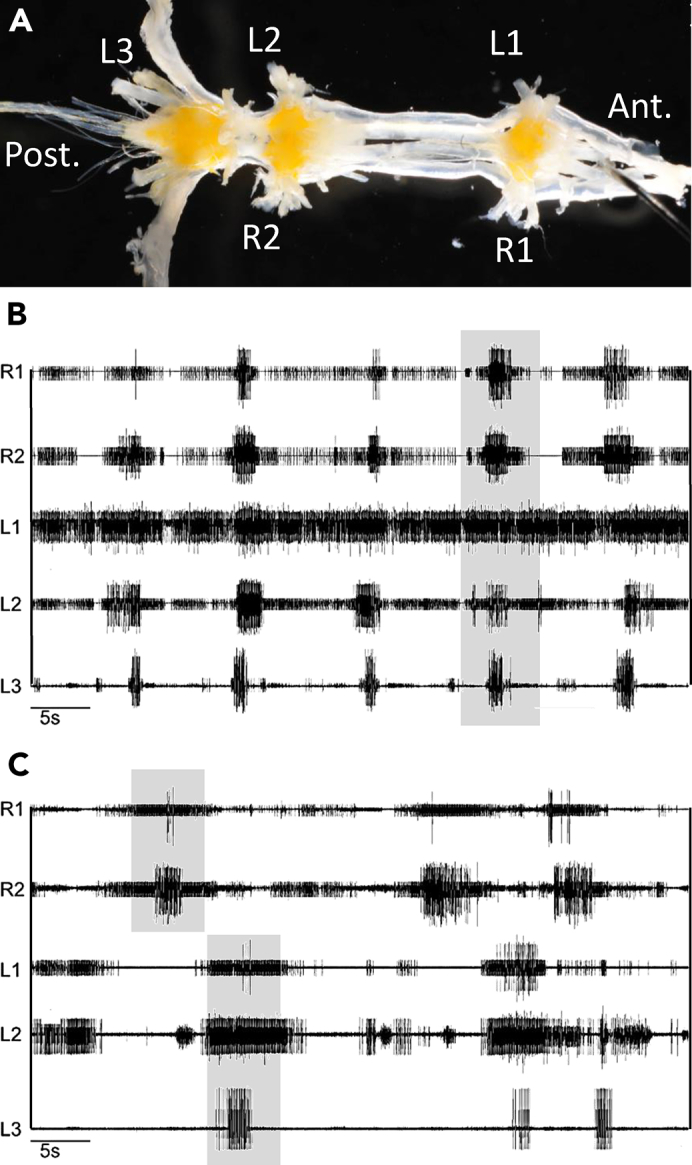
Observing *In Vitro* Locomotive Rhythms Motor patterns recorded from the locust thoracic ganglia preparation *in vitro* (after Knebel et al. [2017]). (A) The isolated thoracic ganglia (from right to left: pro-, meso-, and metathoracic ganglion) with recording sites noted. (B) An example of a recording, after pharmacological activation of the prothoracic ganglion showing synchronized bursts in all recorded nerves. A single instance of such synchronous bursts is highlighted (gray). (C) A sample of a recording after pharmacological activation of the metathoracic ganglion, showing ipsilateral synchronization and bilateral alternation of bursts. R1 and R2: nerves innervating right legs in the first and second thoracic segments of the intact locust; L1–L3: nerves innervating all three left legs in the intact locust.

To address this, in our recent study we conducted a thorough investigation of the central neuronal mechanisms that control leg motor patterns in locust, a leading insect model (Knebel et al., 2017). In this study, we observed the insect's emergent locomotion patterns, by tracking the locomotive rhythms exhibited by the insect's nervous system *in vitro;* namely, we isolated the thoracic nerve chord and measured the activity of the depressor motor neurons following pharmacological activation with the muscarinic agonist pilocarpine (Knebel et al., 2017). We observed three main results (Figure 1): (1) CPGs controlling the left and right legs in the two rostral ganglia (i.e., the pro- and mesothoracic ganglia) have an inherent bilateral synchrony, whereas the CPGs in the caudal, metathoracic ganglion show an anti-phase bilateral preference; (2) each ganglion can recruit the other ganglia to adopt its own bilateral preferred coordination; (3) when all ganglia are activated simultaneously, CPGs in all ganglia tend to show synchronous oscillations, representing a spurious *gait* in which all six legs oscillate as one.

**Figure 2:**
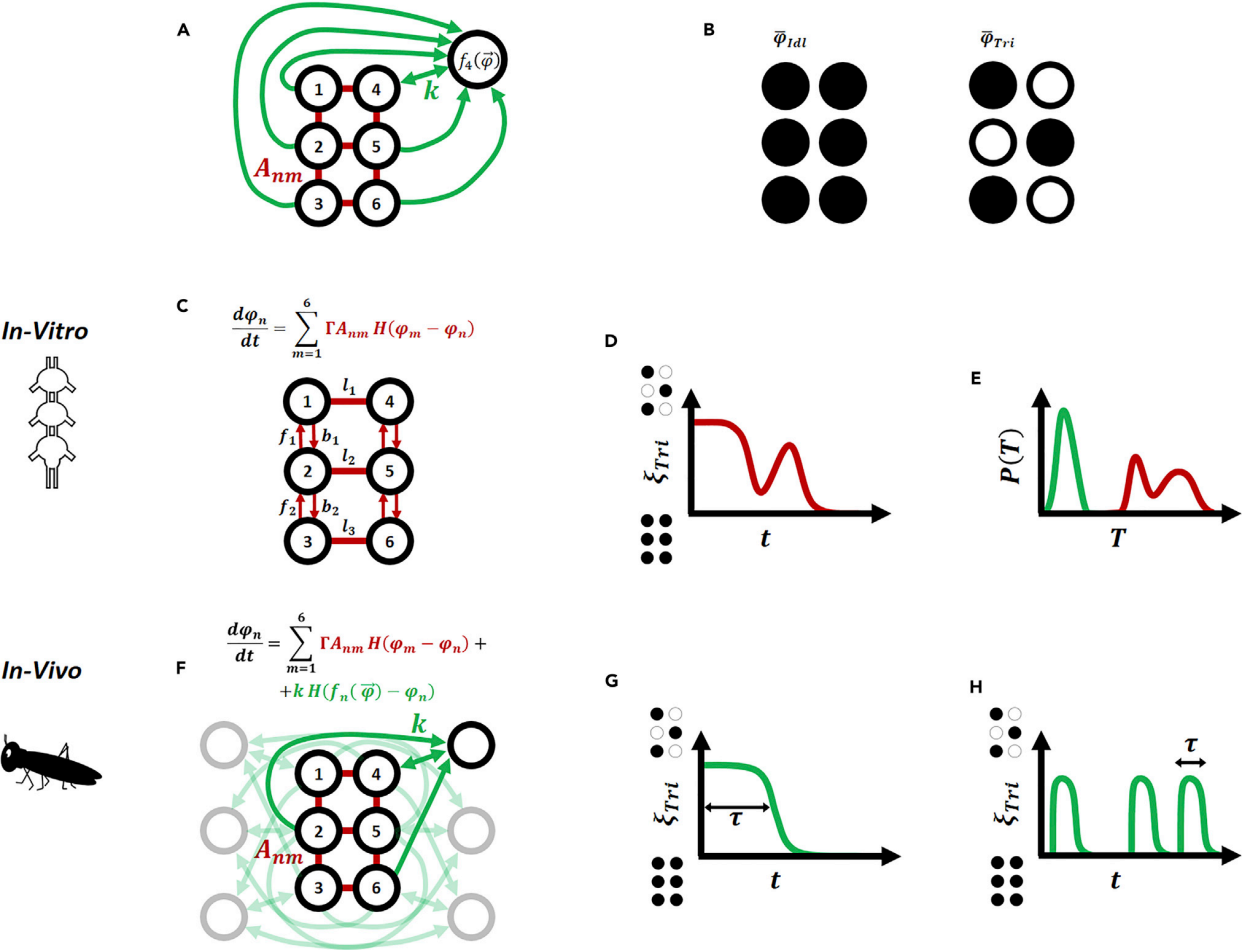
The Ingredients of Insect Locomotion (A) Insect locomotion is captured by the dynamic Equation 1, whose terms capture the physical coupling between all nodes $A_{nm}$ (red) and the dynamic feedback, e.g., sensory input, collected and processed from these nodes (green). Here we show the input $f_{4}\left(\vec{\varphi }\right)$ introduced and processed from all nodes into node 4. (B) The fixed points of Equation 1 represent the potential stable gaits. In idling all legs are in sync (left); in double-tripod the legs split into two anti-phase trios (right). (C) In the *in vitro* preparations sensory feedback is omitted, expressing only the endogenous wiring diagram *A*_*nm*_ between the central pattern generators. *A*_*nm*_ includes 14 directional links, which thanks to the left-right symmetry reduce to 7 independent parameters, *l*_1_,*l*_2_,*l*_3_,*b*_1_,*b*_2_,*f*_1_,*f*_2_. We discuss how to select these parameters in the Transparent Methods section under Supplemental Information. (D) Such isolated preparations do not exhibit a stable double-tripod state, hence upon initiating double-tripod locomotion ($\xi_{\text{Tri}}=1$) the system decays to idling ($\xi_{\text{Tri}}=0$). The observed decay patterns are, however, highly irregular, standing in contrast with the sharp transitions exhibited by live insects. (E) As a result the probability density *P*(*T*) of bout duration is multi-modal, portraying a coexistence of *good* transitions (left peak, green) and *bad* irregular ones (right peaks, red). (F) In live insects each node receives input from its double-tripod counterparts, e.g., node 4 is coupled to nodes 2, 4, and 6. Such feedback helps correct for noise-driven deviations from double-tripod. (G) Under these conditions the double-tripod gait remains unstable; however, it recovers the *in vivo* locomotion patterns: a temporarily stable double-tripod gait, sustained for a typical duration *τ*, followed by a sharp transition to idling. (H) This results in time-limited metastable double-tripod bursts, separated by idling periods of varying duration, concurring with empirically observed locomotion.

These results were obtained, as noted, in the absence of sensory or descending inputs and thus reflect the endogenous wiring diagram of the locust CPG network. Depending on the animal models (Hughes and Wiersma, 1960, Roberts et al., 1998; Wallén and Williams, 1984, Wilson, 1961), this network is assumed to play an important role in shaping locomotion behavior (Ayali et al., 2015a, Ayali et al., 2015b, Büschges et al., 2011). Although our findings agree with patterns observed in other insects (Büschges et al., 1995, Mantziaris et al., 2017), they seem to defy the common perception of the insect's hard-wired locomotive patterns, as, indeed, these recorded motor patterns do not correspond to any functional coordination pattern, or gait, of *in vivo* walking insects. For instance, a common gait demonstrated by many walking insects (including the locust) is the double-tripod gait: two extreme legs on one side are in phase with the middle leg of the other side, and in anti-phase with the other three, resulting in two alternating tripods (Figure 2B, right). This gait is considered extremely stable and is assumed to be partly responsible for the outstanding fast locomotion of some insects and their ability to negotiate different terrains. However, our *in vitro* locust preparations failed to exhibit this locomotion pattern, instead showing synchronous oscillations among all ganglia, suggesting that the locust endogenous network is, in fact, not adapted to enable double-tripod locomotion.

The study described herein was motivated by these discrepancies between the consistent, robust, functional gait seen in walking locust and the non-functional, yet seemingly consistent and stable, coordination patterns observed in the isolated *in vitro* preparations. To settle this disparity we use mathematical models of coupling between CPGs, to uncover the trade-off between the two driving forces of locust locomotion: (1) the natural wiring of the insect's nervous system, which drives it toward synchronous oscillations and (2) the sensory feedback mechanisms, and their processing by higher motor centers, which correct for noise and help sustain temporarily stable bouts of locomotion. The first is present both *in vitro* and *in vivo*, whereas the second is only featured by live insects, explaining the discrepancy between the gaits observed in live insects and isolated preparations. Interestingly, this balance retrieves several frequently encountered features of *in vivo* locomotion, specifically, the time-limited locomotive bouts (Ariel et al., 2014, Bazazi et al., 2012, Kramer and McLaughlin, 2001), whose empirically observed distribution emerges as a natural prediction of our experimentally motivated model.

## Results

### Modeling Locust Locomotion

Mathematical models of insect locomotion are based on data acquired by both observation and experimental manipulation of intact animals and, to a large extent, on fictive motor patterns recorded *in vitro* under isolated, controlled conditions. Early models of insect CPGs used relaxation and delay oscillators (Graham, 1977), with several more detailed descriptions subsequently developed (Cruse, 1990, Schilling et al., 2013). Other approaches span the space of central-decentralized and feedforward-feedback control (Koditschek et al., 2004, Kukillaya et al., 2009, Tóth et al., 2013a, Tóth et al., 2013b), but their complexity makes analysis difficult. Indeed, a *complete* insect model, with 18 joint CPGs, muscles, and sensory pathways would contain over 500 differential equations, severely limiting our ability to extract analytical insight. Hence, here we revert to a phase-reduced model (Proctor and Holmes, 2010) that collapses the complexity of the biophysical details into an effective description of a six-node coupled oscillator network, representing a highly efficient modeling scheme that can be compared directly with data (Ayali et al., 2015b, Borgmann et al., 2011, Fuchs et al., 2012, Ghigliazza and Holmes, 2004, Holmes et al., 2006, Tóth et al., 2015). Within this framework we treat each of the six legs (nodes *n* = 1, …,6) as an oscillator, whose phase *φ*_*n*_(*t*) is driven by

(Equation 1) $$\frac{\text{d}\varphi_{n}}{\text{d}t}=\text{Γ}\sum_{m=1}^{6}A_{nm}H\left(\varphi_{m}-\varphi_{n}\right)+kH\left(f_{n}\left(\vec{\varphi }\right)-\varphi_{n}\right)+\nu \left(t\right)\text{.}$$

The matrix *A*_*nm*_ describes the coupling between the oscillating limbs, whose strength (and sign) is governed by Γ (Figure 2A, red), and *H*(*x*) is a 2*π*-periodic function with *H*(0) = *H*(*π*) = 0 and *H*′(*π*)<0 <*H*′(0). Hence the first term on the right-hand side captures the impact of the insect's endogenous wiring diagram, in the absence of any sensory feedback. To introduce feedback we include the second term on the right-hand side, in which each node receives information on the collective state of all other nodes (Figure 2A, green). This feedback is effectively treated as an additional *virtual* node, whose *state* is captured by $f_{n}\left(\vec{\varphi }\left(t\right)\right)=f_{n}\left(\varphi_{1}\left(t\right),\ldots ,\varphi_{6}\left(t\right)\right)$, a collective function incorporating the instantaneous states of all other oscillators. In fictive locomotion the *in vitro* nervous system is isolated, sensory input is suppressed, and hence *k* = 0. In live insects, on the other hand, we have *k*> 0, allowing each node *n* to constantly monitor its state versus that of the virtual $f_{n}\left(\vec{\varphi }\right)$. The last term $\nu \left(t\right)∼N\left(0,\sigma^{2}\right)$ represents the system's internal noise, a zero-mean Gaussian noise function, in which the noise levels are controlled by the magnitude of the variance $\sigma^{2}$.

The solutions of Equation 1, $\vec{\varphi }\left(t\right)$, describe the instantaneous phases of the oscillating limb, capturing the different gaits exhibited by the insect. Note that in Equation 1 the *frequency* of the oscillations is absent, as, indeed, in realistic gaits, all limbs have identical frequencies, allowing us to transform to the rotating frame, where the common frequency is set to zero. Hence, locomotive gaits are fully characterized by the relative phases, as provided by $\vec{\varphi }\left(t\right)$. For instance, during idling all six limbs have matching phases, hence ${\vec{\varphi }}_{\text{Idl}}=\left(0,0,0,0,0,0\right)$; in contrast, the double-tripod gait is captured by ${\vec{\varphi }}_{\text{Tri}}=\left(0,\pi ,0,\pi ,0,\pi \right)$, an alternating set of phase-shifted trios (Figure 2B).

The fixed gaits featured by the insect can be obtained from Equation 1 by eliminating the noise term and setting the derivative on the left-hand side to zero, namely,

(Equation 2) $$\text{Γ}\sum_{m=1}^{6}A_{nm}H\left(\varphi_{m}-\varphi_{n}\right)+kH\left(f_{n}\left(\vec{\varphi }\right)-\varphi_{n}\right)=0.$$

A dynamically stable gait must also satisfy the linear stability condition, that Equation 1’s Jacobian matrix

(Equation 3) $$\begin{array}{c} J_{nm}=\text{Γ}\left(A_{nm}H^{\prime }\left(\varphi_{m}-\varphi_{n}\right)-\delta_{nm}\sum_{j=1}^{6}A_{nj}H^{\prime }\left(\varphi_{j}-\varphi_{n}\right)\right)\\ +k\left(H^{\prime }\left(f_{n}\left(\vec{\varphi }\right)-\varphi_{n}\right)\left(\frac{\partial f_{n}}{\partial \varphi_{m}}-\delta_{nm}\right)\right)\text{,} \end{array}$$

has a strictly negative real spectrum, namely,

(Equation 4) $$\overset{6}{\underset{i=1}{\text{max}}}\left\{\text{Re}\left(\lambda_{i}\right)\right\}\leq 0\text{,}$$

where *λ*_*i*_ are the eigenvalues of *J*_*nm*_. In Equation 3, the function *H*^′^(*φ*_*m*_−*φ*_*n*_) represents a derivative $H^{\prime }=\partial H/\partial \varphi_{m}$ taken around the fixed point $\vec{\varphi }$; *δ*_*nm*_ is the Kronecker *δ* function.

Each dynamically stable gait $\vec{\varphi }$ must satisfy Equations 2, 3, and 4, providing us with a link between the observed $\vec{\varphi }$ and the parameters of Equation 1. Hence, observing an insect's stable gaits we can retrieve constraints pertaining to the structure and weights of *A*_*nm*_, the magnitudes of Γ and *k*, and the functional form of $f_{n}\left(\vec{\varphi }\right)$. Below, we use this strategy to analyze two empirically observed *gaits*: the synchronous oscillations measured *in vitro* versus the double tripod featured *in vivo*.

### *In Vitro* Fictive Locomotive Rhythms (*k* = 0)

The observed rhythms *in vitro* consistently exhibit stable synchronous oscillations, i.e., ${\vec{\varphi }}_{\text{Idl}}=\left(0,0,0,0,0,0\right)$. These oscillations have non-zero frequency, distinct from stationary idling; however, in the context of Equation 1, where only the phases are important, such synchronous oscillations are indistinguishable from the idling state. As explained above, this empirical observation can help us retrieve information on the terms of Equation 1. Clearly, ${\vec{\varphi }}_{\text{Idl}}$ satisfies the criterion in Equation 2, independent of the specific structure of *A*_*nm*_ or value of Γ, hence its fixed-point status alone provides limited insight. Its observed stability, however, offers meaningful constraints on *A*_*nm*_ and *H*(*x*), which we investigate below. Therefore, we refer to the system's Jacobian matrix in Equation 3, which, for *k* = 0 and $\vec{\varphi }={\vec{\varphi }}_{\text{Idl}}$, takes the form

(Equation 5) $$J_{nm}^{\text{Idl}}=\text{Γ}H^{\prime }\left(0\right)\left[A_{nm}-\delta_{nm}\sum_{j=1}^{6}A_{nj}\right]\text{,}$$

and calculate its six eigenvalues $\lambda_{i}^{\text{Idl}}$, i=1,…,6 (see Transparent Methods under Supplemental Information). The empirically observed stability of ${\vec{\varphi }}_{\text{Idl}}$ *in vitro* suggests that all $\lambda_{i}^{\text{Idl}}$ satisfy Equation 4. Specifically, we have $\lambda_{6}^{\text{Idl}}=-2\text{Γ}H^{\prime }\left(0\right)$, prescribing the condition that

(Equation 6) $$\text{Γ}H^{\prime }\left(0\right)>0.$$

Hence we find that the experimentally observed synchronous rhythms impose constraints on the locust endogenous wiring *A*_*nm*_, Γ, expressed explicitly through Equation 6 and implicitly through $J_{nm}^{\text{Idl}}$’s remaining eigenvalues, i.e., that $\lambda_{1}^{\text{Idl}},\ldots ,\lambda_{5}^{\text{Idl}}$ satisfy Equation 4. The challenge is that, as we next show, these conditions exclude the potential stability of other frequently observed gaits. As an example, let us specifically consider the ubiquitous double-tripod gait ${\vec{\varphi }}_{\text{Tri}}=\left(0,\pi ,0,\pi ,0,\pi \right)$ and examine whether it can coexist with the observed stability of ${\vec{\varphi }}_{\text{Idl}}$. Calculating the Jacobian in Equation 3 around ${\vec{\varphi }}_{\text{Tri}}$, we find

(Equation 7) $$J_{nm}^{\text{Tri}}=\text{Γ}H^{\prime }\left(\pi \right)\left[A_{nm}-\delta_{nm}\sum_{j=1}^{6}A_{nj}\right]=\frac{H^{\prime }\left(\pi \right)}{H^{\prime }\left(0\right)}J_{nm}^{\text{Idl}}\text{,}$$

which provides a direct mapping between $J_{nm}^{\text{Tri}}$ and $J_{nm}^{\text{Idl}}$, and in turn between their corresponding eigenvalues: $\lambda_{i}^{\text{Tri}}=\left[H^{\prime }\left(\pi \right)/H^{\prime }\left(0\right)\right]\lambda_{i}^{\text{Idl}}$. The crucial point is that because $H^{\prime }\left(\pi \right)/H^{\prime }\left(0\right)<0$, the condition in Equation 4 cannot be simultaneously satisfied around both gaits, as, indeed

(Equation 8) $$\text{Re}\left(\lambda_{\text{i}}^{\text{Idl}}\right)\leq 0\Leftrightarrow \text{Re}\left(\lambda_{\text{i}}^{\text{Tri}}\right)\geq 0.$$

Hence, in the absence of feedback, i.e., *k* = 0, ${\vec{\varphi }}_{\text{Idl}}$ and ${\vec{\varphi }}_{\text{Tri}}$ are mutually exclusive stable states of Equation 1.

**Figure 3:**
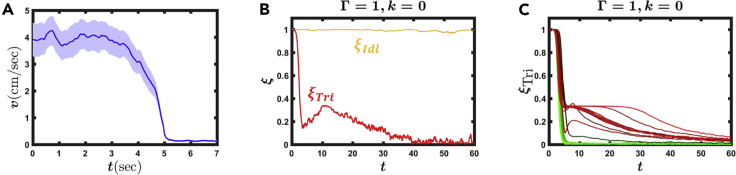
Double Tripod in Reality versus Simulation without Sensory Feedback We averaged 43 real locust locomotion bouts to obtain the profile of a *typical* bout. We then compared this empirical observation to *in vitro* simulations (Equation 1 with k=0). (A) Locust velocity *v* versus time as obtained from the averaged empirical bouts. Bouts feature a stable instance of walking, followed by a sharp transition to idling. Error represents 95% confidence intervals (see Transparent Methods under Supplemental Information). (B) $\xi_{\text{Tri}}\left(t\right)$ (red) and $\xi_{\text{Idl}}\left(t\right)$ (yellow) versus *t* as obtained from Equation 1 with *k* = 0. Idling is stable ($\xi_{\text{Idl}}\left(t\right)\approx 1$), whereas double-tripod gradually breaks down (red). Such transient double-tripod, in and of itself, is consistent with the empirical observations. The challenge is, however, that the pattern of this double-tripod decay is irregular and stretched, as opposed to the empirically observed sharp transitions. (C) A sequence of simulated double-tripod bouts. Approximately half decay sharply, as observed in real insect locomotion (green), whereas the remaining half show irregular decay patterns (red). Together, this demonstrates that real double-tripod locomotion, i.e., stable walking followed by sudden termination, is unattainable without the feedback mechanisms (*k*) of Equation 1. In our simulations we used Γ = 1, *k* = 0, *σ* = 0.01, and *A*_*nm*_ as described in Transparent Methods, under Supplemental Information.

This represents our first key conclusion, driven by the empirically observed synchronous rhythms: that the locust endogenous neuronal network, as described by *A*_*nm*_ and Γ, naturally drives the insect toward idling, and, as a consequence, cannot support a stable double-tripod gait. Therefore, double-tripod can be ignited by a live insect as an initial condition, but in the presence of even the slightest noise *ν*(*t*), it will unconditionally decay back to the naturally stable ${\vec{\varphi }}_{\text{Idl}}$. This conclusion, which at first glance may seem to undermine the premise of insect locomotion, can, in fact, help explain its true nature. Indeed, live locusts do not exhibit stable locomotive gaits, but rather initiate sporadic short bouts of, e.g., double-tripod locomotion, separated by potentially long, stable periods of idling (Ariel et al., 2014, Bazazi et al., 2012, Kramer and McLaughlin, 2001). To observe this, in Figure 3A, we averaged 43 real bouts obtained from the locust, to construct a *typical* empirical double-tripod bout. Indeed, we find that it is best described by a transient state, sharply transitioning to idling after a limited duration.

To confront this observation with Equation 1 we constructed *A*_*nm*_ as appears in Figure 2C, setting Γ = 1. We then tested the dynamics of the system in Equation 1 under no feedback (*k* = 0), starting at t=0 from two initial conditions: $\vec{\varphi }\left(t=0\right)={\vec{\varphi }}_{\text{Idl}}$ and $\vec{\varphi }\left(t=0\right)={\vec{\varphi }}_{\text{Tri}}$. To evaluate the stability of each of these states we measured the order parameters

(Equation 9) $$\xi_{\text{Idl}}\left(t\right)=\frac{1}{6}\left|\sum_{n=1}^{6}e^{i\varphi_{n}\left(t\right)}\right|,\;\;\;\;\;\xi_{\text{Tri}}\left(t\right)=\frac{1}{6}\left|\sum_{n=1}^{6}{\left(-1\right)}^{n}e^{i\varphi_{n}\left(t\right)}\right|\text{,}$$

which range from $\xi_{\text{Idl}}=1$ for a perfect ${\vec{\varphi }}_{\text{Idl}}$ to $\xi_{\text{Idl}}=0$ in the double-tripod regime; similarly we have $\xi_{\text{Tri}}=1$ for a perfect ${\vec{\varphi }}_{\text{Tri}}$ versus $\xi_{\text{Tri}}=0$ as the double-tripod decays to idling. As expected we find that whereas ${\vec{\varphi }}_{\text{Idl}}$ is stable, the double-tripod gait, ${\vec{\varphi }}_{\text{Tri}}$, is unstable, expressed by the gradual decay of $\xi_{\text{Tri}}$ to zero (Figure 3B).

The problem is that while the transient nature of ${\vec{\varphi }}_{\text{Tri}}$ is consistent with the empirically observed locomotive bouts, the temporal profile of this decay is highly unrealistic, in some cases exhibiting a long plateau at $0<\xi_{\text{Tri}}<1$ instead of the empirically observed sharp transition between walking and idling (Figures 3B and 3C, red). This type of locomotion, a continuous period of mixed gaits, is not only physically prohibitive but also stands in sharp contrast with the empirically observed behavior in Figure 3A, where the insect features a sharp transition from walking to idling. Hence we show below that feedback mechanisms play a crucial role in shaping the actual transient profile of the double-tripod gait, leading to the desired abrupt locomotive instances.

### *In Vivo* Locomotive Bouts (k > 0)

To model locomotion in live locust, we enable feedback by setting *k*> 0 on the right-hand side of Equation 1. To sustain a double-tripod gait, the sensory input must mirror to, e.g., node 1 the current phases of nodes 3 and 5, with whom it is supposed to synchronize. This can be achieved through a feedback function of the form

(Equation 10) $$f_{n}\left(\vec{\varphi }\right)=\sum_{m=1}^{6}C_{nm}\varphi_{m}\text{,}$$

where$\sum_{m=1}^{6}C_{nm}=1$ and *C*_*nm*_ = 0 if *n* and *m* are not in the same tripod-trio (see Transparent Methods under Supplemental Information). Using Equation 10 in Equation 1 introduces feedback that reflects to each node the state of its double-tripod counterparts. For instance, node 1 receives feedback on the states of nodes 1, 3, and 5, in the form of a weighted average, with the weights determined by the arbitrary coefficients *C*_*nm*_ (Figure 2F). If 1 diverts from its coordinated motion with 3 and 5, due to internal noise, the information in $f_{n}\left(\vec{\varphi }\right)$ will steer it back toward its desired phase *φ*_1_ = *φ*_3_ = *φ*_5_. Such averaging represents an internal noise correction mechanism, allowing to reinforce the double-tripod gait in the face of naturally occurring disturbances (e.g., ν(t)). In a sense, it serves to re-stabilize the unstable ${\vec{\varphi }}_{\text{Tri}}$. One can also consider an alternative construction, in which the feedback mirrors the counter-tripod limbs, namely, reflect to node 1 the states of nodes 2, 4, and 6, with whom it is supposed to sustain anti-phase oscillations. For simplicity, however, we only examine the positive feedback, i.e., 1, 3, 5 and 2, 4, 6, as described above.

Adding such feedback, the Jacobian in Equation 3 becomes

(Equation 11) $$J_{nm}^{\text{Idl}}=\text{Γ}H^{\prime }\left(0\right)\left[A_{nm}-\delta_{nm}\sum_{j=1}^{6}A_{nj}\right]+kH^{\prime }\left(0\right)\left(C_{nm}-\delta_{nm}\right)\text{,}$$

around ${\vec{\varphi }}_{\text{Idl}}$, and

(Equation 12) $$J_{nm}^{\text{Tri}}=\text{Γ}H^{\prime }\left(\pi \right)\left[A_{nm}-\delta_{nm}\sum_{j=1}^{6}A_{nj}\right]+kH^{\prime }\left(0\right)\left(C_{nm}-\delta_{nm}\right)\text{,}$$

around ${\vec{\varphi }}_{\text{Tri}}$, whose eigenvalue sets, $\lambda_{i}^{\text{Idl}}$ and $\lambda_{i}^{\text{Tri}}$, are shown in the Transparent Methods section under Supplemental Information. We find that for k≳Γ, i.e., corrective feedback comparable in strength to the internal coupling, both Jacobians in Equations 11 and 12 feature five negative (or zero) eigenvalues. Hence their sixth eigenvalue $\lambda_{6}$ is the one that determines the stability of the two states: for $J_{nm}^{\text{Idl}}$ it equals $\lambda_{6}^{\text{Idl}}=-2\text{Γ}H^{\prime }\left(0\right)$, which, following Equation 6 is, indeed, negative. For $J_{nm}^{\text{Tri}}$, on the other hand, we have $\lambda_{6}^{\text{Tri}}=-2\text{Γ}H^{\prime }\left(\pi \right)$. Recalling that *H*^′^(*π*) is opposite in sign to $H^{\prime }\left(0\right)$, we conclude that $\lambda_{6}^{\text{Tri}}$ is inevitably positive, and hence regardless of the strength of the feedback *k*, ${\vec{\varphi }}_{\text{Tri}}$ continues to be unstable.

We have now reached our second key conclusion, that despite feedback, which we explicitly designed to reinforce ${\vec{\varphi }}_{\text{Tri}}$, the double-tripod gait remains unstable. In a sense, we have assumed the *ideal* conditions for double-tripod stability, encoding through Equation 10 an intrinsic *hard-wired* mechanism to correct discrepancies from double-tripod motion, and yet, as long as ${\vec{\varphi }}_{\text{Idl}}$ is stable, double-tripod remains an unstable transient state, independent of the feedback strength *k*. The crucial point is, however, that while in the absence of feedback (*k*→0) the double-tripod bouts exhibit an irregular transient behavior (Figure 3C), the presence of feedback in the form of Equation 10 helps shape them in the desired form of time-limited sharp bursts, as observed in real insect locomotion (Figure 3A).

**Figure 4:**
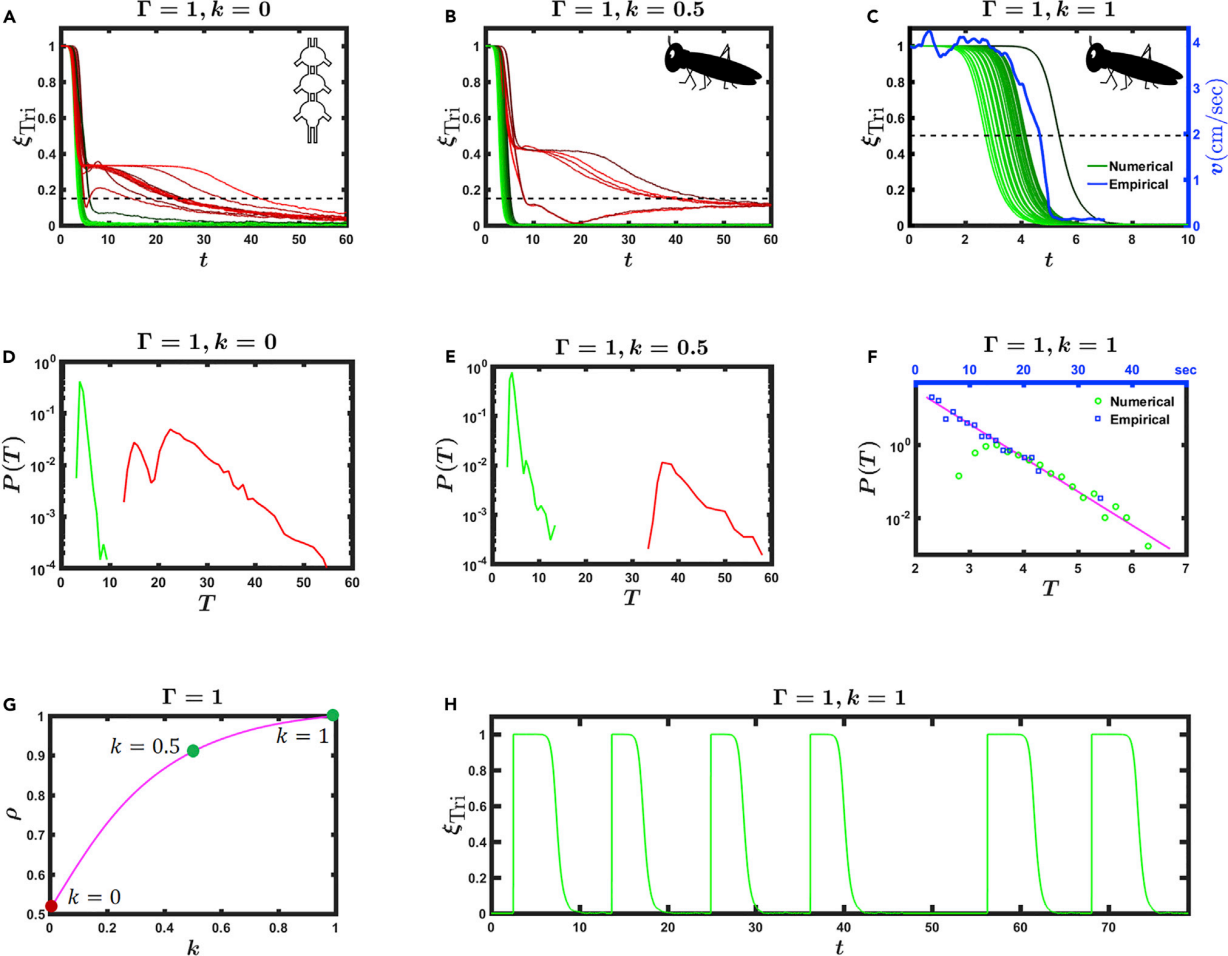
Sharp Locomotive Bouts Shaped by Feedback We tested the patterns of double-tripod locomotive bouts under different levels of sensory feedback *k*. For small *k: k*=0 (A), and *k*=0.5 (B) we continue to observe irregular transitions (red) from double tripod to idling. The number of *bad* transitions decreases as *k* is increased. The duration of each bout is defined at the point at which $\xi_{\text{Tri}}\left(t\right)=0.15$ for the last time, namely, when it crosses the black dashed lines. (C) For *k* = 1 = Γ we obtain perfect bouts (green), featuring a metastable double-tripod gait that terminates with a sudden transition to idling, as observed in empirical patterns of locomotion. Here the duration of each bout is defined as the half-life, marked by the dashed line at $\xi_{\text{Tri}}\left(t\right)=0.5$. For comparison, we also show the average empirical bout *v* versus *t* (also shown in Figure 3A), where we normalized *v*(*t* = 0) = 1 (blue). We find that our simulated bouts successfully retrieve the empirically observed locomotion. (D) The probability density *P*(*T*) versus *T* for a bout of duration *τ*∈(*T*,*T* + d*T*) as extracted from 10^4^ realizations for k=0. A significant fraction of *bad* transitions (red peaks) is observed, corresponding to the long irregular gaits (red) featured in (A). (E) $P(T)$ versus $T$ for $k=0.5$. Irregulat gaits (red peak) continue to be observed. (F) For *k* = 1 = Γ the density *P*(*T*) (green circles) no longer exhibits the *bad* peaks, instead featuring an exponential tail (solid line). This indicates that now all bouts follow a regular bounded form, as observed in (C). We also measured *P*(*T*) from our empirical bouts (blue squares), finding that real locomotion is, indeed, characterized by an exponential distribution, as predicted by our model. Note that in our simulations *T* has arbitrary units (bottom horizontal axis), whereas in the empirical measurements *T* is measured in seconds (top horizontal axis). Therefore, we do not expect the two distributions (green, blue) to fully coincide, only to layout on the same linear slope. (G) Fraction of sharp transitions *ρ* versus *k*. For *k* = 0, i.e., no feedback, *ρ* = 0.5, hence one of every two bouts features an irregular transition; when k≳1=Γ we observe *ρ*→1, representing realistic locomotion patterns. (H) The resulting locomotive bouts for k=1=Γ, featuring regular bounded double-tripod instances separated by varying periods of idling. In our simulations we used Γ = 1, *k* as it appears in each panel, *σ* = 0.01, and *A*_*nm*_ as described in Transparent Methods, under Supplemental Information.

To demonstrate this we repeated in Figure 4 the simulation of Equation 1, this time with varying levels of feedback *k*. Indeed, in the limit of weak feedback, i.e., *k*→0, we continue to observe the non-realistic transitions to idling, a discrepancy occurring in approximately one of every two realizations (Figure 4A). As *k* is increased, however, the frequency of *bad* transitions decreases (Figure 4B), until at k≳Γ we observe perfect metastable double-tripod bouts, all of which have roughly equal duration (Figure 4C, green). Each realization features a clean and stable double-tripod instance, $\xi_{\text{Tri}}\left(t\right)\approx 1$, terminated by a sudden sharp transition to stable idling, $\xi_{\text{Tri}}\left(t\right)=0$. The resulting bouts, indeed, successfully recover the observed structure of the real *in vivo* locomotive bout (blue).

To systematically asses the performance of our model we measured the probability density *P*(*T*) for a tripod bout to have duration *τ*∈(*T*,*T* + d*T*). For *k* = 0 we obtain a multi-modal distribution (Figure 4D), with a bounded density of *good* bouts (green) versus separated peaks of *bad* irregular bouts (red). As *k* is increased the density of irregular bouts decreases (Figure 4E), until at *k*≃Γ it vanishes completely, as predicted (Figure 4F). Under these conditions we find that *P*(*T*) is well fit by an exponential distribution (solid line), indicating that most locomotive bouts are of similar duration. We also extracted *P*(*T*) from our empirically measured sample of locust bouts, finding that it, indeed, features the predicted exponential form (blue squares), an additional independent corroboration for our proposed model. In Figure 4H we show the resulting locomotive bouts (solid line), in which at random instances the locust initiates a double-tripod gait, which then relaxes to idling via Equation 1, exhibiting realistic locomotive patterns.

### Origins of the Metastable Locomotive Bouts

To understand the roots of the observed locomotion patterns, consider the behavior of Equation 1, under the initial condition $\vec{\varphi }\left(t=0\right)={\vec{\varphi }}_{\text{Tri}}$, i.e., double-tripod. Being an unstable state, even the slightest perturbation ${\vec{\varphi }}_{\text{Tri}}+\delta \vec{\varphi }\left(t\right)$, an inevitable consequence of the noise ν(t), will cause the system to divert to the stable ${\vec{\varphi }}_{\text{Idl}}$, hence leading from $\xi_{\text{Tri}}\left(t=0\right)=1$ to $\xi_{\text{Tri}}\left(t\to \infty \right)=0$. The important point, however, is not the transition itself, which is unavoidable, but rather the form of this transition, sharp or irregular. In reality, such perturbations are continuously affecting the system because of the stochastic term *ν*(*t*); however, for simplicity, we consider below the system's response to a single (small) perturbation $\delta \vec{\varphi }\left(0\right)$ introduced at *t* = 0. This allows us to track the evolution of $\delta \vec{\varphi }\left(t\right)$ through the linearized Equation 1, which takes the form

(Equation 13) $$\frac{\text{d}\delta \varphi_{n}}{\text{d}t}=\sum_{m=1}^{6}J_{nm}^{\text{Tri}}\delta \varphi_{m}\left(t\right)\text{,}$$

where$J_{nm}^{\text{Tri}}$ is the system's Jacobian taken from Equation 12. Its solution is

(Equation 14) $$\delta \vec{\varphi }\left(t\right)=\sum_{i=1}^{6}B_{i}{\vec{v}}_{i}\text{exp}\left(\lambda_{i}^{\text{Tri}}t\right)\text{,}$$

where$\delta \vec{\varphi }\left(0\right)=\sum_{i=1}^{6}B_{i}{\vec{v}}_{i}$ is the eigenvector decomposition of the perturbation at *t* = 0 in the base ${\vec{v}}_{i}$ spanned by $J_{nm}^{\text{Tri}}$’s eigenvectors (see Transparent Methods under Supplemental Information). To assess the magnitude of the coefficients *B*_*i*_, we consider the size of the perturbation $\delta \vec{\varphi }\left(0\right)$, which, driven by the system's intrinsic noise levels, *ν*(*t*), has each of its components extracted from $\delta \varphi_{n}\left(0\right)∼N\left(0,\sigma^{2}\right)$. This provides, on average, $\delta \varphi_{n}\left(0\right)∼\pm \sigma$, and hence $|\delta \varphi^{2}\left(0\right)|∼6\sigma^{2}$. Using our eigenvector decomposition, this translates to

(Equation 15) $$\sum_{i=1}^{6}B_{i}^{2}∼6\sigma^{2}\Rightarrow B_{i}∼\sigma \text{,}$$

linking *B*_*i*_ to the system's intrinsic levels of noise. The only exception is *B*_5_, which precedes the eigenvector ${\vec{v}}_{5}=\left(1/\sqrt{6}\right){\left(1,1,1,1,1,1\right)}^{⊤}$, associated with the vanishing $\lambda_{5}^{\text{Tri}}=0$. This vector represents a uniform shift in all phases, having no impact on the gait, which is only characterized by the *relative* phases. Therefore we only focus on perturbations orthogonal to ${\vec{v}}_{5}$, ignoring this trivial *uniform* phase shift, namely, we set *B*_5_ = 0 in Equation 14. We are thus left with only five terms on the right-hand side of Equation 14, *i* = 1, …,4 and *i* = 6 (see Transparent Methods under Supplemental Information).

Let us first analyze the first terms *i* = 1, …,4. For sufficiently strong feedback k≳Γ, we have, for these four terms $\lambda_{i}^{\text{Tri}}<0$, leading to a rapid exponential decay with a typical timescale of *τ*_0_∼*k*^−1^, small in the limit of large *k* (see Transparent Methods under Supplemental Information). This represents a rapid convergence to zero, which leaves Equation 14, after a brief transient time, dominated by the single positive eigenvalue $\lambda_{6}^{\text{Tri}}=-2\text{Γ}H^{\prime }\left(\pi \right)>0$, whose associated eigenvector is ${\vec{v}}_{6}=\left(1/\sqrt{6}\right){\left(1,-1,1,-1,1,-1\right)}^{⊤}$. As a result, Equation 14 converges to

(Equation 16) $$\delta \vec{\varphi }\left(t\right)∼\sigma {\vec{v}}_{6}e^{\frac{t}{\tau_{6}}}\text{,}$$

where

(Equation 17) $$\tau_{6}=\frac{1}{\lambda_{6}^{\text{Tri}}}=-\frac{1}{2\text{Γ}H^{\prime }\left(\pi \right)}\text{,}$$

and where we used Equation 15 to replace *B*_6_ with *σ*. The timescale *τ*_6_, associated with ${\vec{v}}_{6}$, controls the rate of the exponential divergence that drives the system away from the perturbative regime, and toward the stable ${\vec{\varphi }}_{\text{Idl}}$. We, therefore, find that the unstable ${\vec{\varphi }}_{\text{Tri}}$ exhibits, in response to noise, two separate timescales *τ*_0_≪*τ*_6_. The first, *τ*_0_, represents the rapidly decaying components of $\delta \vec{\varphi }\left(t\right)$, driving the system back *toward* ${\vec{\varphi }}_{\text{Tri}}$. Once these short-lived components decay, the system is driven by the remaining component ${\vec{v}}_{6}$, diverging *away* from double-tripod at a rate *τ*_6_.

#### Metastability

Equation 16 describes the temporal behavior of the perturbation $\delta \vec{\varphi }\left(t\right)$, however the true transient profile of the double-tripod bout, and its decay to idling, are captured by $\xi_{\text{Tri}}\left(t\right)$ and $\xi_{\text{Idl}}\left(t\right)$ of Equation 9. Taking the state of system to be ${\vec{\varphi }}_{\text{Tri}}+\delta \vec{\varphi }\left(t\right)$ and extracting $\delta \vec{\varphi }\left(t\right)$ from Equation 16, we can write these two order parameters as (see Transparent Methods under Supplemental Information)

(Equation 18) $$\xi_{\text{Tri}}\left(t\right)=\frac{1}{6}\left|\sum_{n=1}^{6}\text{exp}\left(-i\frac{{\left(-1\right)}^{n}}{\sqrt{6}}\sigma e^{\frac{t}{\tau_{6}}}\right)\right|\approx \frac{1}{6}\left|\sum_{n=1}^{6}\left(1-i\frac{{\left(-1\right)}^{n}}{\sqrt{6}}\sigma e^{\frac{t}{\tau_{6}}}\right)\right|=1\text{,}$$

and

(Equation 19) $$\xi_{\text{Idl}}\left(t\right)=\frac{1}{6}\left|\sum_{n=1}^{6}{\left(-1\right)}^{n}\text{exp}\left(-i\frac{{\left(-1\right)}^{n}}{\sqrt{6}}\sigma e^{\frac{t}{\tau_{6}}}\right)\right|\approx \frac{1}{6}\left|\sum_{n=1}^{6}\left({\left(-1\right)}^{n}-i\frac{1}{\sqrt{6}}\sigma e^{\frac{t}{\tau_{6}}}\right)\right|=\frac{\sigma }{\sqrt{6}}e^{\frac{t}{\tau_{6}}}\text{,}$$

where we have used the linear approximation *e*^−*iɛ*^≈1−*i*ɛ to obtain the estimates on the right-hand side of both expressions. These estimates are valid as long as $\sigma e^{\frac{t}{\tau_{6}}}\ll 1$, or equivalently, as long as *t*≪*τ*, where

(Equation 20) $$\tau =-\tau_{6}\text{ln}\sigma =\frac{1}{2\text{Γ}H^{\prime }\left(\pi \right)}\ln\;\sigma \text{,}$$

and where we have used Equation 17 to express *τ*_6_.

Equations 18, 19, and 20, our final prediction, represent the temporal profile of the transition from an initial double-tripod gait to idling, as predicted by Equation 1. They emerge from the negotiation between the insect's endogenous wiring diagram (*A*_*nm*_, Γ), tending toward idling, and its corrective feedback mechanisms ($k,f_{n}\left(\vec{\varphi }\right)$), designed to reinforce ${\vec{\varphi }}_{\text{Tri}}$ in the face of intrinsic noise ($\delta \vec{\varphi }\left(t\right)$). Most importantly, they provide precisely the desired form of sharp metastable locomotive bouts: Equation 18 describes a plateau of continuous undisturbed double-tripod locomotion, i.e., $\xi_{\text{Tri}}\left(t\right)\approx 1$, whereas Equation 19 exposes the exponential takeover of $\xi_{\text{Idl}}\left(t\right)$, which eventually begins to dominate over $\xi_{\text{Tri}}\left(t\right)$. Finally, Equation 20 predicts the lifetime of the $\xi_{\text{Tri}}\left(t\right)$ plateau, capturing the duration of a typical locomotive bout. Hence, although the feedback cannot force a stable ${\vec{\varphi }}_{\text{Tri}}$, it shapes it in the form of a metastable gait, in which double-tripod locomotion is stably sustained for a limited duration (*τ*) and then sharply transitions to idling—precisely the observed form of the real locomotive bouts.

**Figure 5:**
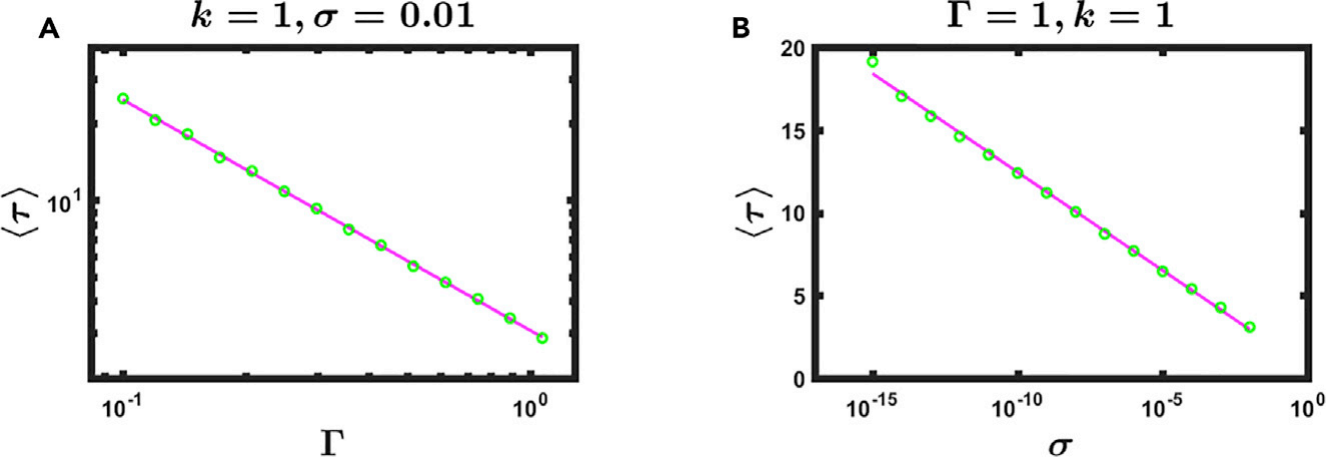
The Lifetime of a Double-Tripod Bout We measured the average duration 〈*τ*〉 (half-life, as in Figure 4C) as obtained from Equation 1 under different coupling strengths Γ and noise levels *σ*. (A) 〈*τ*〉 versus Γ (circles) features the scaling *τ*∼Γ^−1^ (solid line), as predicted by Equation 20. (B) 〈*τ*〉 versus *σ* (circles) shows a logarithmic decline, once again in agreement with Equation 20. In our simulations we used *k* = 1, Γ and *σ* as they appear in each panel, and *A*_*nm*_ as described in Transparent Methods, under Supplemental Information; each data point (circle) represents an average over 100 realizations. For each data point we calculated the error as the 95% confidence interval, namely, Err $\approx 2\mathrm{STD}/\sqrt{n}$, where STD is the standard deviation extracted from the 100 realizations and *n* = 100. The resulting errors are not shown, as they were found to be negligibly small, fitting within the green circles.

To complete the picture, Equation 20 predicts that *τ* is inversely dependent on Γ and scales logarithmically with *σ*. Hence, a perfect noiseless double-tripod gait, i.e., *σ*→0, provides *τ*→∞, a long-lasting burst of locomotion. However, the logarithmic dependence in Equation 20 indicates that even minor noise levels will limit the duration of the actual locomotive intervals. We tested these predictions in Figure 5, finding that, indeed, *τ* scales inversely with Γ and logarithmically with *σ*, in agreement with Equation 20.

## Discussion

Modeling insect locomotion often relies on stable gaits, seeking the parameters in Equation 1 that can offer, e.g., a stable double-tripod state. Here, we have shown, based on empirical observations, that this description must be refined: on the one hand *A*_*nm*_, the insect's internal wiring, is tuned toward idling, ensuring that all instances of locomotive bouts converge to the rest state ${\vec{\varphi }}_{\text{Idl}}$. On the other hand, once ${\vec{\varphi }}_{\text{Tri}}$ is initiated, the insect's encoded feedback mechanisms, Equation 10, correct for noise, and help sustain a temporarily stable locomotive bout. The relative strengths of these two competing forces is captured by the parameters *k* and Γ in Equation 1, whose magnitude determines the role of descending inputs versus that of the internal CPG network. This suggests a spectrum of potential behaviors, from animals whose locomotion is internally wired, i.e., CPG driven, to ones that are controlled by environmental feedback. Our model predicts that the latter will feature extremely weak fictive rhythms *in vitro*, absent sensory feedback, whereas the former will exhibit internally wired gaits, such as ${\vec{\varphi }}_{\text{Tri}}$, even *in vitro*. Encouraging indications in this direction have been recently observed on the stick insect (Ayali et al., 2015a, Mantziaris et al., 2017), which seems to be environment driven, versus the cockroach, which is likely CPG driven (Ayali et al., 2015a, David et al., 2016, Fuchs et al., 2011). Our experiment-based analysis here places the locust between these two extremes with *k* of comparable magnitude to Γ.

This point brings us back to the biology of insect locomotion, and specifically the double-tripod gait. This unique gait is prevalent among all insect models studied thus far: from the slow walking stick insect, where it is mostly observed in young immature animals, to the fast cockroach, where the double-tripod gait is the principal gait used in practically all walking speeds and environmental contexts (Ayali et al., 2015a). Similarly, it was found in moth (Johnston and Levine, 1996), as well as in the fly (Wosnitza et al., 2012). All these different insects likely share basic features of their locomotion wiring diagram (Ayali et al., 2015a), yet they may differ significantly in other attributes, e.g., their intrinsic noise level, or their internal balance between CPGs and sensory feedback. Hence, our modeling approach can provide insight beyond the locust, exposing principles that are generally applicable to insect locomotion. In a broader perspective, metastable states play an important role in many neuronal tasks, which assume a temporary excitation, later relaxing to the globally stable state (Fukai, 1990, Haldeman and Beggs, 2005, Kelso and Tognoli, 2007). Such processes allow organisms to transition to a desired state, e.g., walking, but at the same time avoid fixating at that state for longer than desirable.

Going beyond insects, intermittent motion is a common feature of animal locomotion in general (Kramer and McLaughlin, 2001). Although this intriguing phenomenon has attracted much attention in the behavioral and behavioral ecology fields, this present work is a first attempt to provide a rigorous dynamic model to account for this unique behavior. Last, to the best of our knowledge, intermittent motion as a dominant feature has thus far not been incorporated in bio-inspired technology, and specifically in the growing field of insect-inspired robotics (Aoi et al., 2017, Delcomyn, 2004, Minati et al., 2018, Ritzmann et al., 2000, Schmitt and Holmes, 2001). The current work may provide the means to embark on this and other such interdisciplinary endeavors.

### Limitations of the Study

The experimental data that served as the basis of the current work were obtained from experiments conducted *in vitro*. As previously discussed in much detail (Knebel et al., 2017, Knebel et al., 2019), although this reductionist approach is common and has been very advantageous in the study of neuronal oscillators, there are also some clear limitations to this approach, namely, that cautiousness is advised when directly applying the conclusions to the intact behaving animal. Similarly, this study was based on data extracted exclusively from the locust. Analogous results have been observed using other insect preparations, and, as discussed above, present a case for generalization. Still, as with any such case, oversimplification of the biological complexity should be avoided. Our mathematical analysis condenses the multiple microscopic details of locomotion into a reduced description of phase-coupled oscillators. Although this provides insight into the high-level characteristics of locomotion, e.g., the trade-off between internal and external driving mechanisms, it overlooks the complexity of the microscopic interacting components. Therefore, our modeling framework may provide limited insight on the specific biological *mechanisms* underlying locomotion.

## Data Availability

All codes to reproduce the results presented here are freely accessible at https://github.com/eranreches/The-metastability-of-the-double-tripod-gait-in-locust-locomotion. Experimental data are available upon reasonable request.

## Methods

All methods can be found in the accompanying Transparent Methods supplemental file.

## Supplemental Information

Document S1. Transparent Methods and Figures S1 and S2

## References

[bib1] Aminzare Z., Srivastava V., Holmes P. (2018). Gait transitions in a phase oscillator model of an insect central pattern generator. SIAM J. Appl. Dyn. Syst..

[bib2] Aoi S., Manoonpong P., Ambe Y., Matsuno F., Wörgötter F. (2017). Adaptive control strategies for interlimb coordination in legged robots: a review. Front. Neurorobot..

[bib3] Ariel G., Ophir Y., Levi S., Ben-Jacob E., Ayali A. (2014). Individual pause-and-go motion is instrumental to the formation and maintenance of swarms of marching locust nymphs. PLoS One.

[bib4] Arshavsky Y.I. (2003). Cellular and network properties in the functioning of the nervous system: from central pattern generators to cognition. Brain Res. Brain Res. Rev..

[bib5] Ayali A., Borgmann A., Büschges A., Couzin-Fuchs E., Daun-Gruhn S., Holmes P. (2015). The comparative investigation of the stick insect and cockroach models in the study of insect locomotion. Curr. Opin. Insect Sci..

[bib6] Ayali A., Couzin-Fuchs E., David I., Gal O., Holmes P., Knebel D. (2015). Sensory feedback in cockroach locomotion: current knowledge and open questions. J. Comp. Physiol. A Neuroethol.Sens. Neural Behav. Physiol..

[bib7] Bazazi S., Bartumeus F., Hale J.J., Couzin I.D. (2012). Intermittent motion in desert locusts: behavioural complexity in simple environments. PLoS Comput. Biol..

[bib8] Bender J.A., Pollack A.J., Ritzmann R.E. (2010). Neural activity in the central complex of the insect brain is linked to locomotor changes. Curr. Biol..

[bib9] Borgmann A., Hooper S.L., Büschges A. (2009). Sensory feedback induced by front-leg stepping entrains the activity of central pattern generators in caudal segments of the stick insect walking system. J. Neurosci..

[bib10] Borgmann A., Tóth T.I., Gruhn M., Daun-Gruhn S., Büschges A. (2011). Dominance of local sensory signals over inter-segmental effects in a motor system: experiments. Biol. Cybern..

[bib11] Bucher D. (2009). Neuronal homeostasis: does form follow function or vice versa?. Curr. Biol..

[bib12] Büschges A., Schmitz J., Bässler U. (1995). Rhythmic patterns in the thoracic nerve cord of the stick insect induced by pilocarpine. J. Exp. Biol..

[bib13] Büschges A., Scholz H., El-Manira A. (2011). New moves in motor control. Curr. Biol..

[bib14] Cruse H. (1990). What mechanisms coordinate leg movement in walking arthropods?. Trends Neurosci..

[bib15] Cruse H. (2002). The functional sense of central oscillations in walking. Biol. Cybern..

[bib16] David I., Holmes P., Ayali A. (2016). Endogenous rhythm and pattern-generating circuit interactions in cockroach motor centres. Biol. Open.

[bib17] Delcomyn F. (2004). Insect walking and robotics. Annu. Rev. Entomol..

[bib18] Friesen W.O., Cang J. (2001). Sensory and central mechanisms control intersegmental coordination. Curr. Opin.Neurobiol..

[bib19] Fuchs E., Holmes P., David I., Ayali A. (2012). Proprioceptive feedback reinforces centrally generated stepping patterns in the cockroach. J. Exp. Biol..

[bib20] Fuchs E., Holmes P., Kiemel T., Ayali A. (2011). Intersegmental coordination of cockroach locomotion: adaptive control of centrally coupled pattern generator circuits. Front. Neural Circuits.

[bib21] Fukai T. (1990). Metastable states of neural networks incorporating the physiological dale hypothesis. J. Phys. Math. Gen..

[bib22] Gal R., Libersat F. (2006). New vistas on the initiation and maintenance of insect motor behaviors revealed by specific lesions of the head ganglia. J. Comp. Physiol. A.

[bib23] Ghigliazza R.M., Holmes P. (2004). A minimal model of a central pattern generator and motoneurons for insect locomotion. SIAM J. Appl. Dyn. Syst..

[bib24] Graham D. (1977). Simulation of a model for the coordination of leg movement in free walking insects. Biol. Cybern..

[bib25] Graham D. (1985). Pattern and control of walking in insects, vol. 18 of advances in insect physiology. Adv. Insect Physiol..

[bib26] Guo P., Ritzmann R.E. (2013). Neural activity in the central complex of the cockroach brain is linked to turning behaviors. J. Exp. Biol..

[bib27] Haldeman C., Beggs J.M. (2005). Critical branching captures activity in living neural networks and maximizes the number of metastable states. Phys. Rev. Lett..

[bib28] Holmes P., Full R.J., Koditschek D., Guckenheimer J. (2006). The dynamics of legged locomotion: models, analyses, and challenges. SIAM Rev..

[bib29] Hooper S.L., Weaver A.L. (2000). Motor neuron activity is often insufficient to predict motor response. Curr. Opin.Neurobiol..

[bib30] Hughes G.M., Wiersma C.A.G. (1960). The co-ordination of swimmeret movements in the crayfish, *Procambarus clarkii* (girard). J. Exp. Biol..

[bib31] Johnston R.M., Levine R.B. (1996). Locomotory behavior in the hawkmoth *Manduca sexta*: kinematic and electromyographic analyses of the thoracic legs in larvae and adults. J. Exp. Biol..

[bib32] Kelso J.A.S., Tognoli E. (2007). Toward a Complementary Neuroscience: Metastable Coordination Dynamics of the Brain.

[bib33] Kien J. (1990). Neuronal activity during spontaneous walking—11.correlation with stepping. Comp. Biochem. Physiol. Physiol..

[bib34] Kien J. (1990). Neuronal activity during spontaneous walking—i. starting and stopping. Comp. Biochem. Physiol. Physiol..

[bib35] Kien J., Altman J.S. (1984). Descending interneurones from the brain and suboesophageal ganglia and their role in the control of locust behaviour. J. Insect Physiol..

[bib36] Kien J., Williams M. (1983). Morphology of neurons in locust brain and suboesphageal ganglion involved in initiation and maintenance of walking. Proc. Roy. Soc. Lond. B Biol. Sci..

[bib37] Knebel D., Ayali A., Pflüger H.-J., Rillich J. (2017). Rigidity and flexibility: the central basis of inter-leg coordination in the locust. Front. Neural Circuits.

[bib38] Knebel D., Rillich J., Nadler L., Pflüger H.-J., Ayali A. (2019). The functional connectivity between the locust leg pattern generators and the subesophageal ganglion higher motor center. Neurosci. Lett..

[bib39] Koditschek D.E., Full R.J., Buehler M. (2004). Mechanical aspects of legged locomotion control. Arthropod.Struct. Dev..

[bib40] Kramer D.L., McLaughlin R.L. (2001). The behavioral ecology of intermittent locomotion. Integr. Comp. Biol..

[bib41] Kukillaya R., Proctor J., Holmes P. (2009). Neuromechanical models for insect locomotion: stability, maneuverability, and proprioceptive feedback. Chaos.

[bib42] Mantziaris C., Bockemühl T., Holmes P., Borgmann A., Daun S., Büschges A. (2017). Intra- and intersegmental influences among central pattern generating networks in the walking system of the stick insect. J. Neurophysiol..

[bib43] Marder E., Bucher D. (2001). Central pattern generators and the control of rhythmic movements. Curr. Biol..

[bib44] Marder E., Calabrese R.L. (1996). Principles of rhythmic motor pattern generation. Physiol. Rev..

[bib45] Martin J.P., Guo P., Mu L., Harley C.M., Ritzmann R.E. (2015). Central-complex control of movement in the freely walking cockroach. Curr. Biol..

[bib46] Minati L., Frasca M., Yoshimura N., Koike Y. (2018). Versatile locomotion control of a hexapod robot using a hierarchical network of nonlinear oscillator circuits. IEEE Access.

[bib47] Mu L., Ritzmann R.E. (2008). Interaction between descending input and thoracic reflexes for joint coordination in cockroach: I. descending influence on thoracic sensory reflexes. J. Comp. Physiol. A.

[bib48] Proctor J., Holmes P. (2010). Reflexes and preflexes: on the role of sensory feedback on rhythmic patterns in insect locomotion. Biol. Cybern..

[bib49] Puhl J.G., Mesce K.A. (2010). Keeping it together: mechanisms of intersegmental coordination for a flexible locomotor behavior. J. Neurosci..

[bib50] Ridgel A.L., Ritzmann R.E. (2005). Effects of neck and circumoesophageal connective lesions on posture and locomotion in the cockroach. J. Comp. Physiol. A.

[bib51] Ritzmann R.E., Büschges A. (2007). Adaptive motor behavior in insects. Curr.Opin.Neurobiol..

[bib52] Ritzmann R.E., Quinn R.D., Watson J.T., Zill S.N. (2000). Insect walking and biorobotics: a relationship with mutual benefits. Bioscience.

[bib53] Roberts A., Soffe S.R., Wolf E.S., Yoshida M., Zhao F.-Y. (1998). Central circuits controlling locomotion in young frog tadpoles. Ann. N. Y. Acad. Sci..

[bib54] Schilling M., Hoinville T., Schmitz J., Cruse H. (2013). Walknet, a bio-inspired controller for hexapod walking. Biol. Cybern..

[bib55] Schmitt J., Holmes P. (2001). Mechanical models for insect locomotion: stability and parameter studies. Phys. Nonlinear. Phenom..

[bib56] Skinner F.K., Mulloney B. (1998). Intersegmental coordination in invertebrates and vertebrates. Curr.Opin.Neurobiol..

[bib57] Tóth T.I., Grabowska M., Rosjat N., Hellekes K., Borgmann A., Daun-Gruhn S. (2015). Investigating inter-segmental connections between thoracic ganglia in the stick insect by means of experimental and simulated phase response curves. Biol. Cybern..

[bib58] Tóth T.I., Grabowska M., Schmidt J., Büschges A., Daun-Gruhn S. (2013). A neuro-mechanical model explaining the physiological role of fast and slow muscle fibres at stop and start of stepping of an insect leg. PLoS One.

[bib59] Tóth T.I., Schmidt J., Büschges A., Daun-Gruhn S. (2013). A neuro-mechanical model of a single leg joint highlighting the basic physiological role of fast and slow muscle fibres of an insect muscle system. PLoS One.

[bib60] Wallén P., Williams T.L. (1984). Fictive locomotion in the lamprey spinal cord in vitro compared with swimming in the intact and spinal animal. J. Physiol..

[bib61] Wilson D.M. (1961). The central nervous control of flight in a locust. J. Exp. Biol..

[bib62] Wosnitza A., Bockemühl T., Dübbert M., Scholz H., Büschges A. (2012). Inter-leg coordination in the control of walking speed in drosophila. J. Exp. Biol..

[bib63] Yu X., Friesen W.O. (2004). Entrainment of leech swimming activity by the ventral stretch receptor. J. Comp. Physiol. A.

[bib64] Zill S.N., Keller B.R., Duke E.R. (2009). Sensory signals of unloading in one leg follow stance onset in another leg: transfer of load and emergent coordination in cockroach walking. J. Neurophysiol..

