# Supplementary material for: The Metastability of the Double-Tripod Gait in Locust Locomotion
Source: iScience. 2019 Jan 8;12:53–65. doi: 10.1016/j.isci.2019.01.002 (PMC6352547; doi:10.1016/j.isci.2019.01.002)
Supplement: Document S1. Transparent Methods and Figures S1 and S2 [file mmc1.pdf]

ISCI, Volume *12*

Supplemental Information

The Metastability of the Double-Tripod Gait in Locust Locomotion

Eran Reches, Daniel Knebel, Jan Rillich, Amir Ayali, and Baruch Barzel

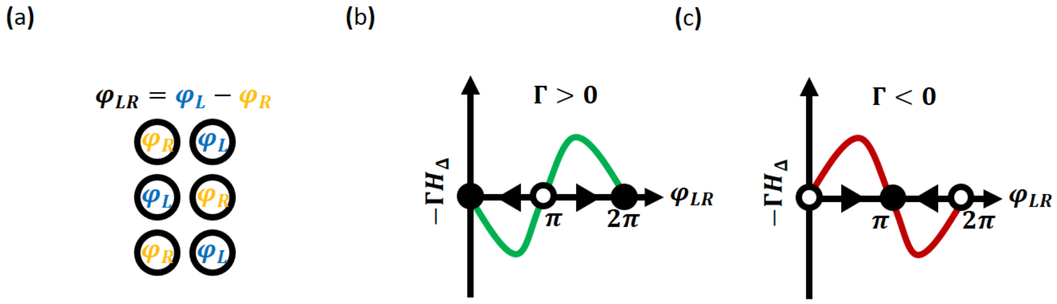

Figure S1. Related to Figure 3b. Reduction to two phase-oscillators. (a) Collapsing the double-tripod gait into two effective oscillators φ_L (blue) and φ_R (orange). The relative phase $\Delta\varphi = \varphi_{LR} = 0$ in idling and π in double-tripod. (b) – (c) The coupling $-\Gamma H_\Delta$ vs. $\Delta\varphi = \varphi_{LR}$ under positive (green) and negative (red) coupling. The dynamically stable gaits appear as solid circles; the unstable ones appear as empty circles. Double-tripod (π) and idling $(0, 2\pi)$ are mutually exclusive stable gaits, the former stable under $\Gamma < 0$, whereas the latter under $\Gamma > 0$.

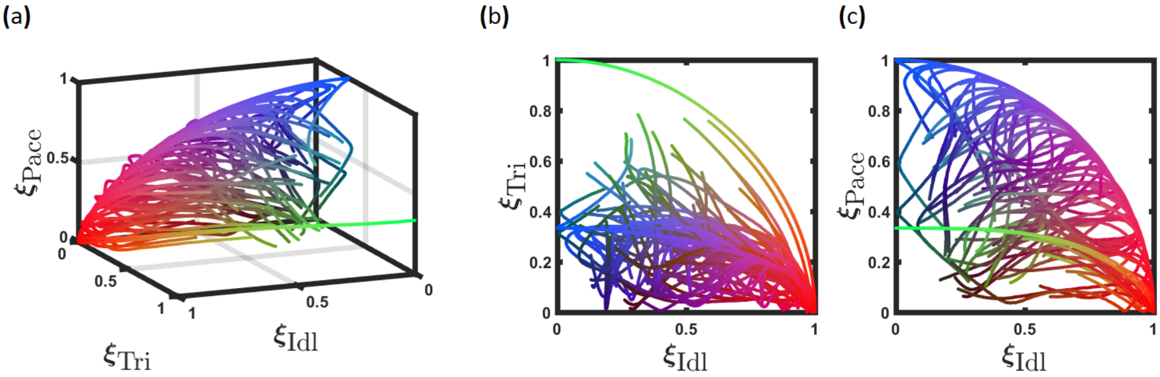

Figure S2. Related to Figure 3b. Stability analysis in gait-space. (a) We selected 100 random initial conditions and mapped their trajectories in the three dimensional *gait*-space. We find that all initial states are drawn to the single basin of attraction, centered around $(1, 0, 0)$, *i.e.* idling. To help follow the paths we use a color code, where $(\xi_{\mathrm{Idl}}, \xi_{\mathrm{Tri}}, \xi_{\mathrm{Pace}})$ are represented by (red, green, blue). (b) – (c) Projections of all trajectories to the two dimensional sub-spaces $(\xi_{\mathrm{Idl}}, \xi_{\mathrm{Tri}})$ and $(\xi_{\mathrm{Idl}}, \xi_{\mathrm{Pace}})$.

Transparent Methods

Network construction. To construct A_{nm} we used the wiring diagram of Fig. 2c, in which there are a total of 14 directed links, allowing us broad degrees of freedom to select all link weights. We then incorporate several constraints that limit these degrees of freedom: (i) Left right symmetry, reduces the independent parameters to seven; (ii) We take the front and middle contralateral links to be identical, *i.e.* $l_1 = l_2$, distinguishing only the back contralateral link. This reduction was indicated by recent experimental observations (Knebel et al. 2017); (iii) As explained below, we normalize the sum of incoming rates to each oscillator (in-degree) to unity (Ghigliazza and Holmes 2004). Together, we remain with three independent parameters in A_{nm}, l_1, l_3 and b_1, providing

$$A_{nm} = \begin{pmatrix} 0 & f_1 & 0 & l_1 & 0 & 0 \\ b_1 & 0 & f_2 & 0 & l_1 & 0 \\ 0 & b_2 & 0 & 0 & 0 & l_3 \\ l_1 & 0 & 0 & 0 & f_1 & 0 \\ 0 & l_1 & 0 & b_1 & 0 & f_2 \\ 0 & 0 & l_3 & 0 & b_2 & 0 \end{pmatrix}, \tag{S1}$$

where $f_1 = 1 - l_1$, $f_2 = 1 - b_1 - l_1$ and $b_2 = 1 - l_3$. In our simulations we set $l_1 = 0.2$, $l_3 = -0.2$ and $b_1 = 0.1$, reflecting the experimental observation that the CPGs controlling the left and right legs in the two rostral ganglia (*i.e.* the pro- and mesothoracic ganglia) have an inherent bi-lateral synchrony, whereas the caudal ones (*i.e.* the metathoracic ganglion) exhibit anti-phase bi-lateral preference (Knebel et al. 2017).

Coupling function and feedback. In our simulations we selected $H(x) = \sin x$, trivially satisfying the required conditions: $H(x)$ is 2π periodic, $H(0) = H(\pi) = 0$ and $H'(\pi) < 0 < H'(0)$. The feedback in Eq. (10) is set to

$$f_{i=1,3,5}\left(\varphi_1, \ldots, \varphi_6\right) = \frac{\varphi_1 + \varphi_3 + \varphi_5}{3} \tag{S2}$$

$$f_{i=2,4,6}\left(\varphi_1, \ldots, \varphi_6\right) = \frac{\varphi_2 + \varphi_4 + \varphi_6}{3}, \tag{S3}$$

reflecting to each node the average phase over its double-tripod counterparts, in effect driving the node towards its desired phase, helping sustain the double-tripod gait in the face of noise.

Spectrum analysis. Under these conditions, the eigenvalues of the Jacobian matrix in Eq. (3) are: around $\vec{\varphi}_{\rm Idl}$

$$\lambda_1^{\rm Idl} = -kH'(0) + \Gamma H'(0)\left[l_1 + \frac{l_3}{2} - \frac{3}{2} + \frac{\sqrt{4b_1(l_3-l_1)+(l_3-1)^2}}{2}\right]$$

$$\lambda_2^{\rm Idl} = -kH'(0) + \Gamma H'(0)\left[l_1 + \frac{l_3}{2} - \frac{3}{2} - \frac{\sqrt{4b_1(l_3-l_1)+(l_3-1)^2}}{2}\right]$$

$$\lambda_3^{\rm Idl} = -kH'(0) + \Gamma H'(0)\left[-l_1 - \frac{l_3}{2} - \frac{1}{2} + \frac{\sqrt{4b_1(l_3-l_1)+(l_3-1)^2}}{2}\right]$$

$$\lambda_4^{\rm Idl} = -kH'(0) + \Gamma H'(0)\left[-l_1 - \frac{l_3}{2} - \frac{1}{2} - \frac{\sqrt{4b_1(l_3-l_1)+(l_3-1)^2}}{2}\right]$$

$$\lambda_5^{\rm Idl} = 0$$

$$\lambda_6^{\rm Idl} = -2\Gamma H'(0),$$

and around $\vec{\varphi}_{\rm Tri}$

$$\lambda_1^{\rm Tri} = -kH'(0) + \Gamma H'(\pi)\left[l_1 + \frac{l_3}{2} - \frac{3}{2} + \frac{\sqrt{4b_1(l_3-l_1)+(l_3-1)^2}}{2}\right]$$

$$\lambda_2^{\rm Tri} = -kH'(0) + \Gamma H'(\pi)\left[l_1 + \frac{l_3}{2} - \frac{3}{2} - \frac{\sqrt{4b_1(l_3-l_1)+(l_3-1)^2}}{2}\right]$$

$$\lambda_3^{\rm Tri} = -kH'(0) + \Gamma H'(\pi)\left[-l_1 - \frac{l_3}{2} - \frac{1}{2} + \frac{\sqrt{4b_1(l_3-l_1)+(l_3-1)^2}}{2}\right]$$

$$\lambda_4^{\rm Tri} = -kH'(0) + \Gamma H'(\pi)\left[-l_1 - \frac{l_3}{2} - \frac{1}{2} - \frac{\sqrt{4b_1(l_3-l_1)+(l_3-1)^2}}{2}\right]$$

$$\lambda_5^{\rm Tri} = 0$$

$$\lambda_6^{\rm Tri} = -2\Gamma H'(\pi).$$

• *Eigenvalues* $\lambda_1^{\rm Tri}, \ldots, \lambda_4^{\rm Tri}$. The first four eigenvalues around $\vec{\varphi}_{\rm Tri}$ have the following structure: k is multiplied by the negative $-H'(0)$ and Γ is multiplied by a product of two terms, the negative $H'(\pi)$ and the function $Z_i(b_1, l_1, l_3)$ $(i, 1, \ldots, 4)$ comprising the elements of A_{nm}. For example, in $\lambda_1^{\rm Tri}$ we have $Z_i(b_1, l_1, l_3) = l_1 + l_3/2 - 3/2 + \sqrt{4b_1(l_3-l_1)+(l_3-1)^2}/2$. With this structure it is guaranteed that if $Z_i \geq 0$ then $\lambda_i^{\rm Tri} < 0$. Under these conditions $\vec{\varphi}_{\rm Tri}$ is stable with respect to perturbations in the $\vec{v}_i$ direction. If however $Z_i < 0$, the corresponding

eigenvalue is still negative if

$$k > \Gamma \frac{H'(\pi)}{H'(0)} Z_i(b_1, l_1, l_3). \tag{S4}$$

The fraction on the right hand side is of order unity, being exactly one in case $H(x) = \sin x$. The last term Z_i is also typically of order unity, since all its components b_1, l_1, l_3, are extracted from A_{nm}, which is normalized such than $\sum_{m=1}^{6} A_{nm} = 1$. Hence, excluding extreme cases, most of A_{nm}'s entries, and therefore also $Z_i(b_1, l_1, l_3)$, are of the order one. It thus follows that $\lambda_i^{\text{Tri}} < 0$ for $i = 1, \dots, 4$ as long as $k \gtrsim \Gamma$, as appears in the main text • *Eigenvalue* λ_5^{Tri}. This eigenvalue corresponds to the constant eigenvector $\vec{v}_5 = (1, 1, 1, 1, 1, 1)^\top/\sqrt{6}$. A perturbation in the direction of $\vec{v}_5$ represents a uniform shift in all phases, having no effect on the relative phases, and hence has no bearing on state of the system. Such perturbation, which does not affect the insect's gait, should not grow or decay in time, but remain constant, as indeed ensured by the fact that $\lambda_5^{\text{Tri}} = \lambda_5^{\text{Idl}} = 0$. • *Eigenvalue* λ_6^{Tri}. Since λ_6^{Idl} is negative, as, indeed $\vec{\varphi}_{\text{Idl}}$ is stable, we have $\Gamma H'(0) > 0$, and hence $\Gamma H'(\pi) < 0$ - consequently, $\lambda_6^{\text{Tri}} > 0$. Therefore, following an arbitrary perturbation $\delta\vec{\varphi}(0)$, all components in the direction of $\vec{v}_1, \dots, \vec{v}_4$ decay exponentially, the $\vec{v}_5$ component has no effect, and the system's departure from the double-tripod gait is governed by $\vec{v}_6 = (1, -1, 1, -1, 1, -1)^\top/\sqrt{6}$ at a rate determined by λ_6^{Tri}.

Normalization of C_{nm} in $f_n(\vec{\varphi})$. As explained above, the dynamics Eq. (1), must be invariant under a uniform phase shift $\delta\vec{\varphi} \propto (1, 1, 1, 1, 1, 1)^\top$, as such shift conserves all relative phases between the oscillators. This is only ensured if $\lambda_5^{\text{Tri}} = \lambda_5^{\text{Idl}} = 0$. Therefore, both Jacobian matrices in Eqs. (11) and (12) must satisfy $J \cdot \vec{v}_5 = 0$, mapping to $\sum_{m=1}^{6} J_{nm} = 0$ (where we use J to denote J^{Idl} or J^{Tri}, respectively). This, in turn, leads to $\sum_{m=1}^{6}(C_{nm} - \delta_{nm}) = 0$, and hence to the normalization condition $\sum_{m=1}^{6} C_{nm} = 1$.

Dimension reduction (Fig. S1). To specifically analyze the behavior of Eq. (1) around $\vec{\varphi}_{\text{Idl}}$ and $\vec{\varphi}_{\text{Tri}}$ we use the unique symmetries of these gaits to reduce the six-dimensional description to a single equation. Consider $\Delta\varphi = f_1(\vec{\varphi}) - f_2(\vec{\varphi})$, where $f_i(\vec{\varphi})$ are taken from Eqs. (S2) and (S3). This parameter captures the relative phases between the two tripod-trios, being $\Delta\varphi = 0, 2\pi$ for idling and $\Delta\varphi = \pi$ for double-tripod. Summing over the relevant equations, we use Eq. (1) to construct a direct equation for $\Delta\varphi$ (under $k = 0$), obtaining

$$\frac{\mathrm{d}\Delta\varphi}{\mathrm{d}t} = -\Gamma H_\Delta(\Delta\varphi), \tag{S5}$$

where $H_\Delta(\Delta\varphi) = H(\Delta\varphi) - H(-\Delta\varphi)$. The stable fixed points satisfy $H_\Delta(\Delta\varphi) = 0$ and $-\Gamma H'_\Delta(\Delta\varphi) < 0$. Since $H(x)$ and $H'(x)$ are both 2π periodic we have $H(j\pi) = H(-j\pi)$ for all $j = 0, 1, \ldots,$ providing $H'_\Delta(0) = 2H'(0)$ and $H'_\Delta(\pi) = 2H'(\pi)$. We, therefore, recover the stability criteria: for idling we have $-2\Gamma H'(0) < 0$ and for double-tripod we require $-2\Gamma H'(\pi) < 0$. As explained in the main text, these represent two mutually exclusive conditions.

The basin of attraction of the idling gait (Fig. S2). The two competing states we consider - $\vec{\varphi}_{\rm Idl}$ vs. $\vec{\varphi}_{\rm Tri}$ – are characterized by unique symmetries that allow rigorous analytical treatment, either as we do in the paper, or through the reduction to a two-phase system. Most generally, however, it is difficult to analyze the complete six-phase system, and the reduction to two phases is not necessarily relevant, absent the special symmetries of $\vec{\varphi}_{\rm Idl}$ and $\vec{\varphi}_{\rm Tri}$. Hence, in principle, there might be an additional stable fixed-point $\vec{\varphi}$ besides $\vec{\varphi}_{\rm Idl}$, that our analysis overlooks. To examine this, as a first step, we tested stability, specifically for unique gaits, such as pace or gallop, finding that they, too are unstable. This, of course, cannot exclude the potential stability of any arbitrary state $\vec{\varphi}$. On the other hand, most arbitrary phase vectors $\vec{\varphi}$ do not constitute a meaningful gait anyhow, and hence even if they are stable, it seems that they are likely not featured by real insects. Still, for completeness, we used numerical simulations to examine the phase-space, and test for the existence of additional stable fixed-points. To reduce the six-dimensional space to a presentable two or three-dimensions, we consider three gaits: $\vec{\varphi}_{\rm Idl} = (0,0,0,0,0,0)^T$, $\vec{\varphi}_{\rm Tri} = (0,\pi,0,\pi,0,\pi)^T$ and $\vec{\varphi}_{\rm Pace} = (0,0,0,\pi,\pi,\pi)^T$, which we characterize by

$$\xi_{\rm Idl}(t) = \frac{1}{6}\left|\sum_{n=1}^{6} e^{i\varphi_n(t)}\right|, \quad \xi_{\rm Tri}(t) = \frac{1}{6}\left|\sum_{n=1}^{6}(-1)^n e^{i\varphi_n(t)}\right|,$$

$$\xi_{\rm Pace}(t) = \frac{1}{6}\left|\sum_{n=1}^{3} e^{i\varphi_n(t)} + \sum_{n=4}^{6}(-1)^n e^{i\varphi_n(t)}\right|, \tag{S6}$$

all ranging from zero, when the gait is absent, to unity for a perfect idling/double-tripod/pace gait. Starting from an arbitrary (six-dimensional) state $\vec{\varphi}$ we track the trajectory of the system in the reduced three-dimensional gait-space $(\xi_{\rm Idl}, \xi_{\rm Tri}, \xi_{\rm Pace})$. We find in Fig. S2 that all such trajectories lead to $(1,0,0)$, *i.e.* a perfect idling state. Had there been another basin of attraction $\vec{\varphi}$, one would expect that some of the initial conditions

would lead to it, which in gait-space would be expressed by an arbitrary mixture (a, b, c). Here we studied 100 random initial conditions, including also the specific initial conditions of a perfect double-tripod, given by $(\xi_{\rm Idl}, \xi_{\rm Tri}, \xi_{\rm Pace}) = (0, 1, 1/3)$ and a perfect pace, *i.e.* $(\xi_{\rm Idl}, \xi_{\rm Tri}, \xi_{\rm Pace}) = (0, 1/3, 1)$. As the figure indicates, we find no other attractor than $\vec{\varphi}_{\rm Idl}$, namely $(1, 0, 0)$ in gait-space. Note that while our initial conditions span the entire six-dimensional phase-space, in this reduced gait-space some areas are avoided. For instance, one cannot have, for any arbitrary selection of $\vec{\varphi}$, the points $(1, 1, 1)$ or $(0, 0, 0)$ in this gait-space, therefore our initial conditions and their ensuing trajectories systematically avoid the corners of this space. Also, while in the complete six dimensional space trajectories cannot intersect, in the projection to gait-space, such intersections are possible. Together, this analysis indicates that idling is indeed the unique steady state of the system. We emphasize again that, while excluding any other gait is not possible analytically, and hence our reliance on numerical analysis, the specific exclusion of the double-tripod stability is, in fact, an analytical result, independent of the specific choice of parameters.

Numerical simulations. To simulate the behavior of Eq. (1) we used an Euler stepper. The stochastic term was treated using the Euler-Maruyama scheme, in which $\nu(t)$ is generated via $\mathrm{d}\nu = X\sigma\sqrt{\mathrm{d}t}$, where $X \sim \mathcal{N}(0, 1)$ is a Normally distributed random variable, and $\mathrm{d}t$ is the differential time-step of the Euler algorithm.

The order parameters $\xi_{\rm Idl}$ and $\xi_{\rm Tri}$. To derive $\xi_{\rm Idl}$ and $\xi_{\rm Tri}$ in Eqs. (18) and (19) we introduce the perturbed state $\vec{\varphi}_{\rm Tri} + \delta\vec{\varphi}(t)$ into Eq. (9). For $\xi_{\rm Tri}$ we have

$$\xi_{\rm Tri} = \frac{1}{6}\left|\sum_{n=1}^{6}(-1)^n e^{i\varphi_n} e^{i\delta\varphi_n(t)}\right|. \tag{S7}$$

Using $\vec{\varphi}_{\rm Tri} = (\pi, 0, \pi, 0, \pi, 0)^\top$, we have $e^{i\varphi_n} = (-1)^n$, which together with the existing factor of $(-1)^n$ in Eq. (S7) leads to $\xi_{\rm Tri} = 1/6|\sum_{n=1}^{6} e^{i\delta\varphi_n(t)}|$. Next we use Eq. (16), with $\vec{v}_6 = (1/\sqrt{6})(1, -1, 1, -1, 1, -1)^\top$, to arrive at the result of Eq. (18). A similar derivation leads to $\xi_{\rm Idl}$ in Eq. (19).

Empirical bouts. To quantitatively measure walking bouts in living locusts, 50 animals were placed in a round arena (diameter of 60 cm). A video camera monitored their activity from above. Subsequently, manual tracking of the locusts was conducted for ~ 6 minutes

of the movie. The distance moved between subsequent frames was used for estimating the locusts' instantaneous speed, allowing, at 29 frames-per-second, a temporal resolution of $\sim 3 \times 10^{-2}$ seconds. Walking termination profile was calculated as follows. Walking was defined as a movement exceeding 0.5 cm/s. Only walking bouts longer than 5 seconds followed by a pause of at least 2 seconds were analyzed, providing a total of 43 individual bouts. The speed profiles of all selected bouts were calculated and overlaid by fixing their pause onsets across each other (first frame in which the speed crossed 0.5 cm/s at the end of the bout). In this alignment all bouts terminate concurrently, allowing us to meaningfully capture the (average) profile of the termination. To obtain the plot of Fig. 3a the profiles were averaged at each time point t. To estimate the error at t we used 95% confidence intervals, Err $\approx 2\sigma(t)/\sqrt{n(t)}$, where $\sigma(t)$ is the standard deviation of the sample at time t and $n(t)$ is the size of each sample, here being $n(t) = 43$ for all t. To measure $P(T)$ in Fig. 4f (blue), we used a more comprehensive set of empirical bouts, to accumulate a sufficient statistical sample. Hence, we collected all walking bouts for which $\tau \geq 1$sec, separated by a pause of similar duration. This results in s sample of 307 bouts, ranging from ~ 1 to 35 seconds.

Ghigliazza, R. M. and Holmes, P. (2004). A minimal model of a central pattern generator and motoneurons for insect locomotion, *SIAM Journal on Applied Dynamical Systems* **3**(4): 671–700.

Knebel, D., Ayali, A., Pflüger, H.-J. and Rillich, J. (2017). Rigidity and flexibility: The central basis of inter-leg coordination in the locust, *Frontiers in Neural Circuits* **10**: 112.
